# Central retinal vein occlusion following intravitreal injections: a case series highlighting multifactorial risk

**DOI:** 10.1186/s40942-025-00781-3

**Published:** 2025-12-15

**Authors:** Michael Antonietti, Carolina Mercado, William E. Smiddy, Stephen G. Schwartz

**Affiliations:** 1 Department of Ophthalmology, Massachusetts Eye and Ear Infirmary, Harvard Medical School, Boston, USA; 2 Bascom Palmer Eye Institute, 3880 Tamiami Trail N, Naples, FL 34103 USA; 3 Bascom Palmer Eye Institute, University of Miami Miller School of Medicine, Miami, USA

**Keywords:** Central retinal vein occlusion (CRVO), Intravitreal injections, Age-Related macular degeneration (AMD), Vascular risk factors, Brolucizumab, Pegcetacoplan, Ranibizumab

## Abstract

**Introduction:**

Intravitreal injections are standard treatment for various manifestations of age-related macular degeneration (AMD) but are associated with rare complications, including central retinal vein occlusion (CRVO). This report describes three cases of CRVO following intravitreal injections in patients with AMD.

**Methods:**

Retrospective case series describing three patients with AMD who developed CRVO after intravitreal injections.

**Results:**

CRVO occurred after ranibizumab (Lucentis, South San Francisco, CA), brolucizumab (Beovu, Novartis, Basel, Switzerland), and pegcetacoplan (Syfovre, Apellis, Waltham, MA) injections. All patients had multiple systemic vasculopathic risk factors. No intraocular inflammation or vasculitis was observed on examination, and fluorescein angiography was not performed. Visual outcomes were poor despite anti-VEGF treatment. None of the three patients developed neovascular glaucoma under our observation. The timing relationship between injection and CRVO varied, and causality could not be established.

**Conclusion:**

CRVO may occur after intravitreal injections across different drug classes, even with agents that have low inflammatory potential. These cases highlight the multifactorial nature of this event and the importance of considering systemic vasculopathy when evaluating post-injection complications.

## Introduction

Intravitreal injections have become standard therapy in treating age-related macular degeneration (AMD) and other retinal disorders, becoming one of the most commonly performed ophthalmic procedures [1]. However, intravitreal injections are associated with risks such as endophthalmitis, retinal detachment, and intraocular pressure (IOP) elevation [2]. An association with arterial or venous occlusive events has been reported, especially in patients with pre-existing vascular risk factors [3]. Central retinal vein occlusion (CRVO) is a rare but serious complication, that warrants further attention, especially in patients with predisposing vascular risk factors. We report three cases of patients who developed CRVO following intravitreal injections, their outcomes, and management.

## Methods

This is a retrospective, consecutive case series of three patients with CRVO after intravitreal injections treated at the University of Miami, Bascom Palmer Eye Institute, Miami, FL. Institutional Review Board approval was obtained from the University of Miami Miller School of Medicine Sciences Subcommittee for the Protection of Human Subjects, and informed consent was waived due to the use of retrospective data. The research adhered to the Tenets of the Declaration of Helsinki.

## Case presentation

### Case 1

**Fig. 1:**
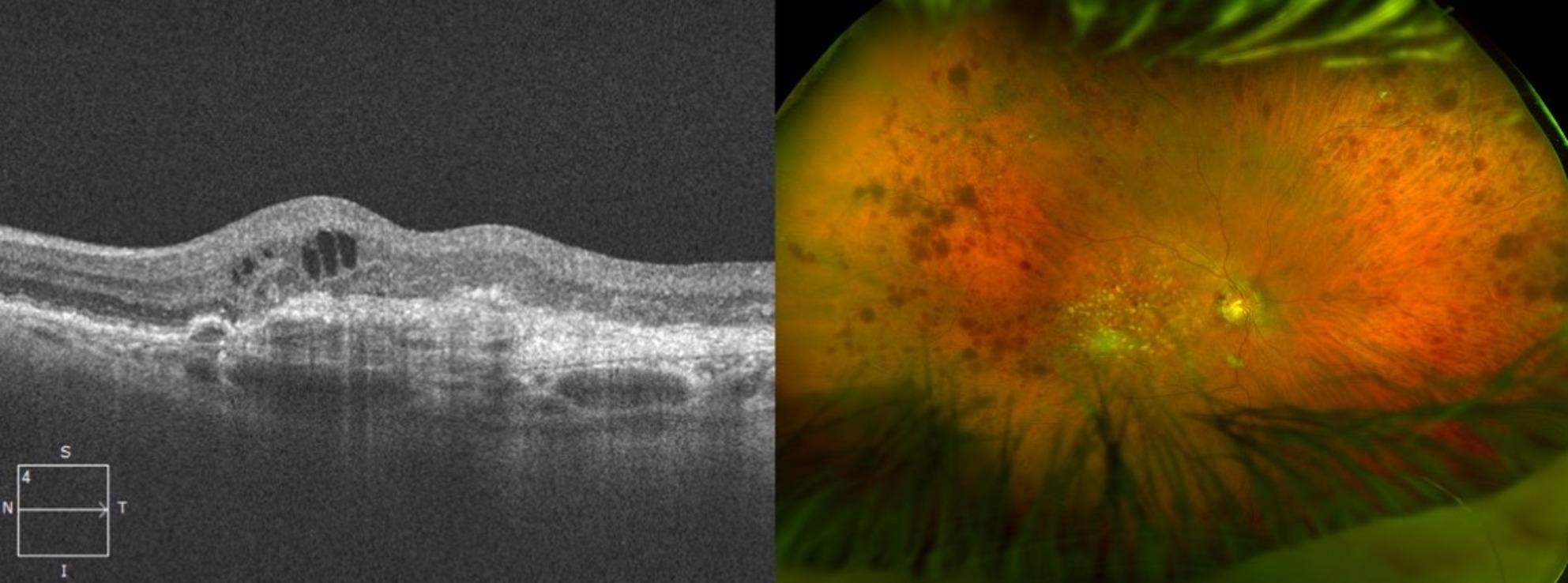
**a**) Optical coherence tomography (OCT) of the right eye’s macula shows thickening of the inner and outer retinal layers, with associated intraretinal fluid. **b**) Fundus photo denoting central retinal vein occlusion of the right eye of a patient who received ranibizumab

An 84-year-old male presented for evaluation of worsening vision OD despite treatment with ranibizumab for neovascular AMD OU elsewhere. His medical history was significant for hypertension, dyslipidemia, and skin melanoma undergoing systemic chemotherapy. There was no prior diagnosis of glaucoma in this patient. He reported treatment with monthly ranibizumab OU, although the number of previous injections is unknown. Best corrected visual acuity (BCVA) OD was 20/250, a decline from his baseline of 20/30. Examination was notable for (CRVO) plus evidence of neovascular AMD OD (Fig. 1). He reported a gradual decline in vision beginning shortly after his most recent ranibizumab injection, with CRVO becoming clinically apparent within approximately 3–4 weeks; however, the exact onset could not be determined because injections were performed outside our institution. The management plan involved continuing monthly ranibizumab injections OU. The IOP remained normal after the presentation and the patient did not develop neovascular glaucoma under our observation. The patient was scheduled for a cardiology evaluation and carotid ultrasound. The patient returned to his original ophthalmologist (in another state) and was lost to our follow-up.

### Case 2

**Fig. 2:**
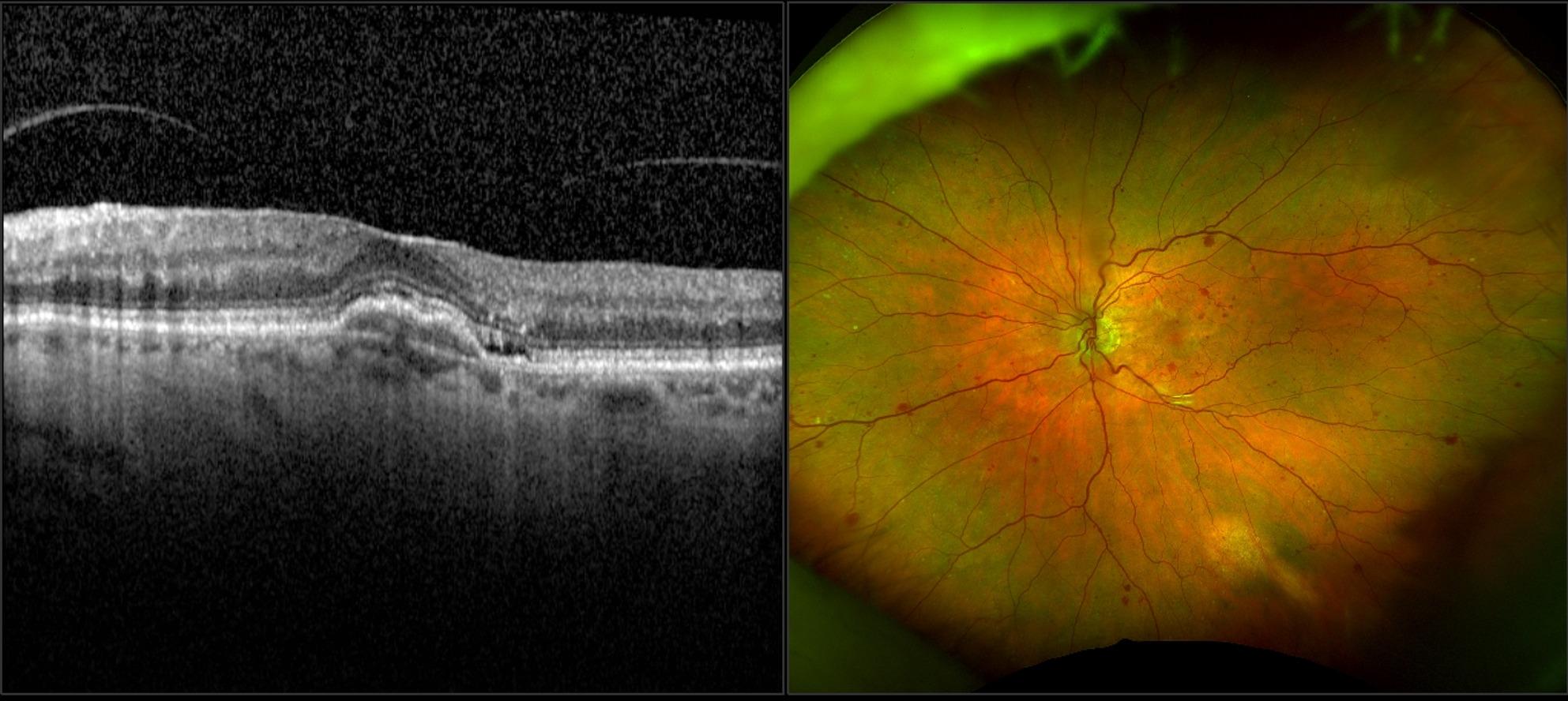
**a**) Optical coherence tomography (OCT) of the left eye’s macula showing paracentral acute middle maculopathy and subretinal fluid. **b**) Fundus photo denoting central retinal vein occlusion of the left eye of a patient who received brolucizumab

An 80-year-old female with neovascular AMD OU was treated with aflibercept (Eylea, Regeneron, Tarrytown, NY) OU, then switched to brolucizumab for three monthly injections in an attempt to extend treatment intervals. Her medical history was significant for type 2 diabetes mellitus, hypertension, and obstructive sleep apnea. There was no prior diagnosis of glaucoma in this patient. Following the third brolucizumab injection, she noted the sudden appearance of a ‘gray map’ in her vision within the first week, and CRVO was diagnosed during that same interval. On examination she was noted to have CRVO OS (Fig. 2). There was no evidence of intraocular inflammation or vasculitis. The patient was switched back to aflibercept OU. Despite continued treatment, BCVA OS declined from 20/25 (prior to the third brolucizumab injection) to 20/400 OS. The patient did not develop neovascular glaucoma under our observation. She became lost to our follow-up 16 months following the third brolucizumab injection.

### Case 3

An 86-year-old female with quiescent neovascular AMD, previously treated with off-label bevacizumab (Avastin, Genentech, South San Francisco, CA), aflibercept, and faricimab (Vabysmo, Genentech, South San Francisco, CA) was noted to have progressive macular atrophy OU. Anti-VEGF injections were stopped and pegcetacoplan injections were initiated OU.

**Fig. 3:**
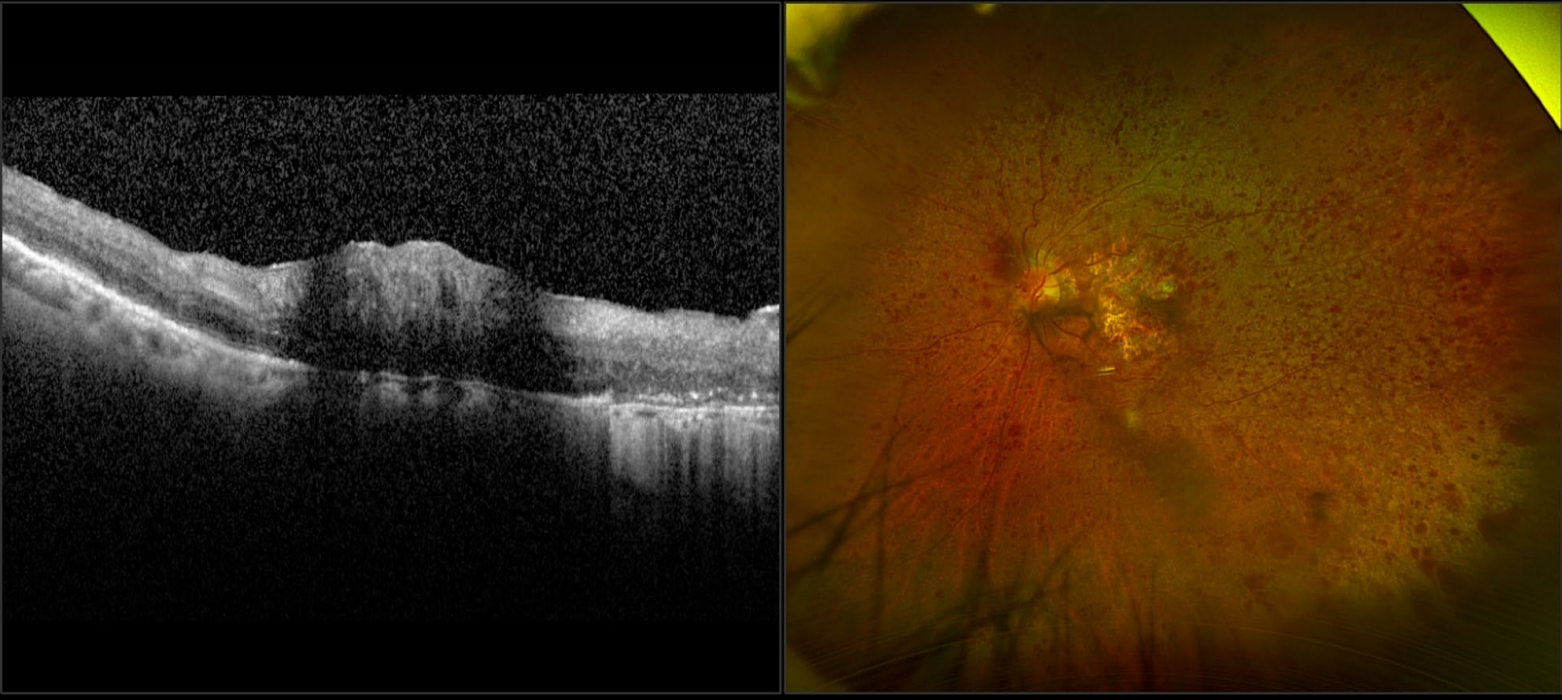
**a**) Optical coherence tomography (OCT) of the left eye’s macula showing paracentral acute middle maculopathy, intraretinal fluid, and disruptions of the external limiting membrane. **b**) Fundus photo denoting central retinal vein occlusion of the left eye of a patient who received pegecetacoplan

Her past medical history was significant for hypertension and dyslipidemia. There was no prior diagnosis of glaucoma in this patient. Following her third pegcetacoplan injection (administered at two-month intervals), she developed a gradual decline in vision beginning soon after the injection, and CRVO was diagnosed within approximately 4 weeks. Her vision continued to worsen progressively during this interval without a discrete sudden drop. On examination CRVO OS was noted (Fig. 3). Pegcetacoplan injections were stopped OU, and faricimab injections were re-started OS. By 5 months following the third pegcetacoplan injection, BCVA OS declined from 20/80 to 20/300. The patient did not develop neovascular glaucoma under our observation. The patient subsequently became lost to our follow-up.

## Discussion

This small, retrospective, nonconsecutive case series identified three patients who developed CRVO without evidence of severe vasculitis or intraocular inflammation following intravitreal injections for AMD (Table 1). The temporal relationship varied across cases: one patient developed CRVO within the first week after injection, while the other two experienced onset within the following month. Two patients developed CRVO after an anti-VEGF injection for neovascular AMD and another after a complement inhibitor injection for macular atrophy. All patients had many risk factors for CRVO including hypertension, dyslipidemia, diabetes mellitus, cancer, and sleep apnea. None of the three patients had a prior diagnosis of glaucoma, and none developed neovascular glaucoma under our observation.

**Table 1:** Overview of patient demographics, ocular history, systemic risk factors, intravitreal agent exposure, temporal relationship between injection and CRVO onset, clinical course, and outcomes in three cases of post-injection CRVO

Characteristic	Case 1	Case 2	Case 3
Age / Sex	84-year-old male	80-year-old female	86-year-old female
Eye Affected	OD	OS	OS
Relevant Ocular History	Neovascular AMD OU	Neovascular AMD OU	Neovascular AMD with macular atrophy OU
Systemic Comorbidities	Hypertension, dyslipidemia, melanoma on chemotherapy	Diabetes mellitus type 2, hypertension, sleep apnea	Hypertension, dyslipidemia
Intravitreal Agent at Time of Event	Ranibizumab (unknown dose)	Brolucizumab (third dose)	Pegcetacoplan (third dose)
Injection Interval / Setting	Monthly; injections performed outside institution	Monthly x3	Every 2 months x3
Onset of Symptoms Relative to Injection	Gradual decline beginning shortly after injection; CRVO apparent within 3–4 weeks	Sudden “gray map” within first week	Gradual decline beginning soon after injection; CRVO diagnosed within 3–5 weeks
Management	Continued ranibizumab; referred back to primary retina specialist	Switched back to aflibercept	Pegcetacoplan stopped; faricimab restarted
Visual Outcome	Declined from 20/30 to ~ 20/250 OD	Declined from 20/25 to 20/400 OS	Declined from 20/80 to 20/300 OS
Neovascular Glaucoma	None	None	None
Follow-Up	Lost to follow-up	Lost to follow-up	Lost to follow-up

The pathophysiology of CRVO is multifactorial, but is generally considered to be caused by reduction of blood flow through the central retinal vein, often by thrombus, at or just posterior to the lamina cribrosa. Major risk factors include increased age, vasculopathy risk factors (systemic hypertension, hyperlipidemia, smoking), and elevated IOP or glaucoma [4–6]. Ischemic CRVO is reported to be more common in patients with glaucoma [7]. IOP elevations may occur acutely following an intravitreal injection [8] and are reported to be more common with rapid injection techniques and high injection volumes [9]. In addition, some patients receiving a series of intravitreal injections develop elevated baseline IOP over time [10]. This ocular hypertension may be considered a predisposing factor to CRVO after intravitreal injections.

Anti-VEGF agents alter vascular permeability and endothelial function in animal models [11–14]. Vascular adverse events, including CRVO and occlusive vasculitis, have been reported in patients treated with anti-VEGF agents [3]. Reduced vessel peak systolic and end-diastolic flow has been reported on central retinal artery doppler ultrasonography following bevacizumab injections, whereas other investigators have found no evidence of worsening ischemia on fluorescein angiography after injections [13, 14]. In general, vasoconstriction has been noted to be more frequent in arteries than veins [3, 15]. 

More recently, brolucizumab and pegcetacoplan have been associated with intraocular inflammation including retinal vascular occlusions [16–19]. The etiology of occlusive vasculitis observed with both drugs remains unknown. It is hypothesized that higher baseline levels of anti-drug antibodies (ADAs) are associated with intraocular inflammation in patients treated with brolucizumab. For pegcetacoplan, a synthetic pegylated drug, the presence of anti-PEG antibodies has been linked to inflammation with other pegylated drugs and may contribute to the complications observed with pegcetacoplan [18]. A clinicopathologic report of two eyes that developed occlusive retinal vasculitis following pegcetacoplan suggested a mixed-type, delayed hypersensitivity reaction [20]. While inflammation has been proposed as a mechanism in some cases, the inclusion of a patient with CRVO following ranibizumab, an agent with a well-established low inflammatory profile, indicates that inflammation alone cannot fully explain these events. This suggests that CRVO following intravitreal injections is likely multifactorial, with systemic vascular comorbidities, transient IOP elevations, and coincidental vascular events in high-risk individuals serving as important contributors. Thus, the overall message of this report is that CRVO may occur rarely after intravitreal injections across different drug classes, and clinicians should maintain awareness of this possibility particularly in patients with multiple vasculopathic risk factors. Notably, fluorescein angiography was not performed in any of the three patients, limiting the ability to definitively exclude subtle vasculitis.

The growing body of evidence linking certain intravitreal agents to rare but serious complications, such as retinal vasculitis, has prompted some clinicians to reevaluate their therapeutic choices; most have decreased the use of brolucizumab and some have reduced use of pegcetacoplan. The rate for all kinds of vascular events after intravitreal injections remains relatively low (0.01%), and a higher rate is seen in diabetic patients (2.6%).^3^ Ranibizumab has demonstrated a favorable vascular safety profile in the ANCHOR [21] and MARINA [22] trials, which followed 280 and 477 patients respectively for two years of repeated intravitreal ranibizumab, and no retinal vascular events were reported. In contrast, pegcetacoplan is associated with rare cases of occlusive vasculitis, with a post-marketing vascular event rate of 0.01% across all injections, but a higher rate of approximately 1:4000 (0.025%) specifically with the first injection. Brolucizumab has been associated with a comparatively higher frequency of inflammatory complications; in the HAWK and HARRIER studies, 36 of 50 eyes with intraocular inflammation (3.3%) exhibited retinal vasculitis and 23 (2.1%) developed concomitant retinal vascular occlusion [23]. These findings underscore the potential risks associated with these agents.

A temporal association between these two events does not confirm drug-related or injection-related toxicity. The clinical examinations did not show any signs of intraocular inflammation, including anterior chamber reaction, vitreous cells, or evidence of progressive disease. To our knowledge, these are the first cases of isolated CRVO reported in patients who received brolucizumab and pegcetacoplan excluding the patients included in the HAWK/HARRIER and OAKS/DERBY trials [23, 24]. 

Interpreting these cases requires caution. It is challenging to know if all these events were caused by the intravitreal injection procedure (with increased IOP), medication toxicity, or some other cause. Because the three patients received three different agents, each with its own safety profile and potential risk factors, the number of variables makes it unlikely that a single mechanism applies to all cases. It is also possible that different mechanisms contributed in different patients. Even though anti-VEGF agents are the standard treatment for CRVO, these cases suggest that anti-VEGF therapy does not prevent CRVO in patients with underlying vasculopathic risk factors.

Another limitation is the uncertainty in the timing between the most recent injection and the onset of CRVO in some of the cases. For patients who were treated outside our institution, documentation was incomplete, and the exact onset relative to the injection could not be precisely determined. This is particularly relevant in the first case, where the patient had longstanding neovascular AMD and a gradual decline in vision; the CRVO may have developed earlier than recognized.

In the second case, the patient had recently received brolucizumab, a drug known to carry a higher risk of retinal vasculopathy compared with older anti-VEGF agents. A drug-related mechanism is therefore possible, although no intraocular inflammation was detected. The marked drop in visual acuity is not fully explained by the available imaging, which limits interpretation.

In the third case, the patient had macular atrophy from neovascular AMD rather than non-neovascular geographic atrophy, which complicates assessment of visual outcomes.

A final limitation is the absence of fluorescein angiography in all three patients. Without angiographic evaluation, subtle retinal or choroidal vascular inflammation, small-vessel occlusions, or perfusion abnormalities could not be excluded. This restricts the ability to determine whether these CRVOs were entirely non-inflammatory or whether there may have been mild vasculopathic or inflammatory findings not visible on clinical examination alone.

Taken together, these factors highlight that while CRVO may occur after intravitreal injections, establishing causation is difficult. The multifactorial nature of these cases including systemic vasculopathic risk factors, drug-specific considerations, incomplete timing data, and limited imaging should be acknowledged when interpreting these findings. These cases underscore the need for careful documentation and imaging when CRVO is suspected following intravitreal therapy, particularly with newer agents.

In conclusion, isolated cases of CRVO without inflammation or vasculitis may occur after intravitreal injections in patients with baseline risk factors. These cases illustrate that the complication is not limited to agents with known inflammatory potential, but may also occur with low-inflammatory agents such as ranibizumab, underscoring the multifactorial nature of this event. Clinicians must weigh the benefits of therapy against the rare risk of CRVO, particularly in patients with systemic vasculopathy, and should counsel patients accordingly.

## Data Availability

No datasets were generated or analysed during the current study.
